# Eliminating Neglected Tropical Diseases in Urban Areas: A Review of Challenges, Strategies and Research Directions for Successful Mass Drug Administration

**DOI:** 10.3390/tropicalmed3040122

**Published:** 2018-11-21

**Authors:** Alayne M. Adams, Myriam Vuckovic, Eleanor Birch, Tara A. Brant, Stephanie Bialek, Dahye Yoon, Joseph Koroma, Abdel Direny, Joseph Shott, Jean Frantz Lemoine, Massitan Dembele, Margaret C. Baker

**Affiliations:** 1 Department of International Health, Georgetown University, Reservoir Road NW, Washington, DC 20057, USA; mv225@georgetown.edu (M.V.); emb263@georgetown.edu (E.B.); dcy7@georgetown.edu (D.Y.); 2 Centers for Disease Control and Prevention, 1600 Clifton Road, Atlanta, GA 30329, USA; twb9@cdc.gov (T.A.B.); zqg7@cdc.gov (S.B.); 3 Family Health International 360 (FHI360) Ghana Country Office, 1st Floor Marvel House 48A Giffard Road, Accra, Ghana; JKoroma@fhi360.org; 4 RTI International, 701 13th St NW, Suite 750, Washington, DC 20005, USA; adireny@rti.org; 5 Office of Infectious Diseases, Global Health Bureau, U.S. Agency for International Development, 1300 Pennsylvania Ave NW, Washington, DC 20352, USA; jshott@usaid.gov; 6 Institutional affiliation: Ministry of Health and Population, Delmas 60, Rue Lemercier #2, Port-au-Prince, Haiti; tileum@hotmail.com; 7 Ministry of Health Mali, BP: 233, Bamako, Mali; masdembele@yahoo.fr

**Keywords:** neglected tropical diseases, mass drug administration, urban health

## Abstract

Since 1950, the global urban population grew from 746 million to almost 4 billion and is expected to reach 6.4 billion by mid-century. Almost 90% of this increase will take place in Asia and Africa and disproportionately in urban slums. In this context, concerns about the amplification of several neglected tropical diseases (NTDs) are warranted and efforts towards achieving effective mass drug administration (MDA) coverage become even more important. This narrative review considers the published literature on MDA implementation for specific NTDs and in-country experiences under the ENVISION and END in Africa projects to surface features of urban settings that challenge delivery strategies known to work in rural areas. Discussed under the thematics of governance, population heterogeneity, mobility and community trust in MDA, these features include weak public health infrastructure and programs, challenges related to engaging diverse and dynamic populations and the limited accessibility of certain urban settings such as slums. Although the core components of MDA programs for NTDs in urban settings are similar to those in rural areas, their delivery may need adjustment. Effective coverage of MDA in diverse urban populations can be supported by tailored approaches informed by mapping studies, research that identifies context-specific methods to increase MDA coverage and rigorous monitoring and evaluation.

## 1. Introduction

Between 1950 and 2014, the global urban population grew from 746 million to almost 4 billion and is expected to reach 6.4 billion by mid-century. Almost 90% of this increase will take place in Asia and Africa [1]. The stark inequities produced by rapid urbanization are well established and most visible in informal settlements, where low-quality housing, crowding and multiple adverse exposures increase health risk, yet access to formal health services is limited. In these contexts, some experts have expressed concerns about the amplification of certain neglected tropical diseases and suggest that special efforts are needed to achieve effective mass drug administration (MDA) coverage in certain urban settings [2,3].

Affecting more than 1 billion people globally, neglected tropical diseases (NTDs) comprise a group of 20 parasitic, bacterial and viral infections [4] that cause considerable morbidity, primarily in marginalized and impoverished populations living in the tropics and subtropics. A subset are considered ‘preventive chemotherapy and transmission control’ or PCT NTDs as they can be controlled or eliminated through periodic MDA campaigns in eligible populations without requiring individual diagnosis [5]. These include lymphatic filariasis (LF), onchocerciasis, schistosomiasis, trachoma and the soil-transmitted helminth infections (STHs; roundworms, hookworms and whipworms), which account for more disability adjusted life-years than malaria. Their health effects include substantial disability ranging from skin and mucous membrane irritation to disabling lymphedema and hydrocele, organ damage, blindness and growth and cognitive deficits, and have contributed to large-scale lost productivity, stigma and discrimination [3].

To globally achieve NTD control and prevention goals, it is imperative that appropriate doses of preventive chemotherapy reach a high proportion of the at-risk population annually. However, many countries have experienced challenges in achieving target MDA coverage in at least three known hard-to-reach populations—notably, urban dwellers, people living in insecure areas and minority populations [6]. Additionally, in many countries other less well characterized groups may be systematically missed or routinely non-compliant [7]. 

In light of rapid urbanization, reaching at-risk populations in urban contexts is a priority concern for NTD programs globally, especially with respect to LF and STH infections, as well as onchocerciasis and schistosomiasis in peri-urban settings. A 2010 survey of African NTD program managers highlighted the lack of urban-specific guidance from the World Health Organization on mapping and treating urban areas [8]. Challenges with program implementation in urban areas has also been reported by the NTD programs supported by the U.S. Agency for International Development (USAID)–the ENVISION and END in Africa projects. For example, in Nepal the national program conducted six rounds of MDA for LF but struggled to get the required coverage in the urban area of Katmandu Valley [9]. In Haiti, while much of the country has achieved stopping targets for LF, five communes of the metropolitan area of Port au Prince with persistently low coverage will need to continue MDA for the foreseeable future at substantial program cost [10]. Similar challenges with MDA coverage in urban areas have been confronted by other national programs including Nepal, Mali, Indonesia and Nigeria [9]. In this narrative review we consider unpublished national program experiences and the limited published literature to highlight urban-specific implementation challenges as well as promising strategies to increase MDA coverage in these settings. Many of these strategies are based on reports from program implementors as few pre-post evaluations of programmatic adaptations are available. Emerging from this discussion is a proposed research agenda to more effectively direct urban MDA investments towards globally endorsed disease control and prevention or elimination objectives.

## 2. Challenges in Implementing NTD Programs in Urban Settings

A narrative review of the literature on MDA implementation and the experiences of NTD programs supported by the ENVISION and END in Africa projects revealed several features unique to urban areas that challenge MDA delivery strategies known to work in rural areas. Among these are the complexities of urban governance, population heterogeneity, mobility and difficulties related to building community trust in these settings. 

### 2.1. Complex Urban Governance

In urban settings, ambiguities in the respective mandates of national, provincial and municipal governments are common and stakeholder interests are many. Shifting political alliances and often unclear responsibilities for district versus municipal jurisdictions may challenge efforts to identify the most appropriate officials to be engaged in MDA. These issues often occur in a context of competition for scarce resources.

Governance challenges may be amplified in peri-urban areas where urban population growth spills into adjacent rural areas. Administrative procedures and capacity to support MDA for specific NTDs will differ depending on whether the activities fall within municipal jurisdiction or some form of district-level authority. Failure to understand the roles and responsibilities of respective authorities and effectively coordinate between different agencies providing services, may hamper NTD program effectiveness [11].

An absence of clear and effective urban governance may also challenge ambitions for program sustainability. Currently, several major pharmaceutical companies have committed to medicine donations to control NTDs in endemic countries, representing the largest public-private partnership program in the world. Recent systematic reviews report that the cost of MDA for LF ranges from $0.06 to $2.70 per person protected per year [12,13]. With rates of urban population growth in Africa in excess of 3.5% per year, the implementation costs of MDA are also projected to increase. Thus, as populations grow, achieving MDA coverage targets as quickly as possible becomes even more urgent. Efforts to diversify and increase support for MDA programing in NTD affected regions are further hindered in contexts of overstretched urban health financing, governance and implementation capacity. 

Another governance challenge inherent to work in urban areas is law and order, the absence of which may compromise the security of MDA personnel and participants. Some slum settlements have high rates of crime and violence and may sequester criminal elements distrustful of outsiders. In the poor urban areas of Santo Domingo in the Dominican Republic, for instance, gang activity and armed violence posed particular risks to MDA workers unfamiliar with local realities [14]. 

The informality of many of these settlements may also hamper the delivery of MDA. Local maps are often unavailable and government investments in roads and basic infrastructure for health and social services tend to be minimal [15]. In campaigns utilizing door-to-door delivery strategies, these realities frustrate efforts to find and treat everyone. NTD elimination programs that require multiple rounds of treatment, may be especially compromised in these circumstances. 

### 2.2. Population Heterogeneity

Urban populations are highly diverse in terms of their social and economic characteristics and living and working conditions. All too often this heterogeneity creates fault lines along which inequities in NTD risk and access to prevention and treatment services become apparent. For example, among residents of informal settlements lacking adequate shelter or access to safe water, hygiene and/or sanitation, exposure to NTDs may be greater than those living in more affluent areas who are better positioned to invest in hygiene and preventive measures such as mosquito screens, bed nets and latrines. At the same time, the wealthy can be hard to reach with MDA interventions. For example, in Ghana, community drug distributors did not feel comfortable working in high-income residential areas, or offices and institutions [16]. Similarly, in Indonesian cities, multi-unit, high-rise apartments occupied by the better-off, posed challenges for MDA distributors [17] given their anonymity and size. 

Differences in the nature of social interaction may also impact MDA. Compared to rural areas, many urban settings are characterized by anonymity and a higher fluctuation of residents within any one neighborhood. As a result, there may be fewer opportunities for community members to interact and less possibility to nurture engagement and information exchange about MDA activities. Weaker extended family connections and greater engagement of women in the workforce, may also limit the potential flow of information about health and health services [11].

Heterogeneity may also be apparent in perceptions of NTD risk comparing urban dwellers of different socioeconomic strata. For example, Njomo et al. linked low treatment coverage among higher socioeconomic groups with their tendency to eschew preventive action, given lower perceived risk and greater perceived access to healthcare [18]. Similarly, lower rates of compliance were found in high-income groups in Pondicherry in southern India [19], attributed in part to perceptions that preventive medicine is not vital when treatment is possible and affordable. 

### 2.3. Mobility

Urban populations tend to be more mobile than those in rural areas as many residents work and live in different parts of the city or change addresses frequently for reasons of convenience and cost. In low- and middle-income countries, circular migration between rural and urban areas is also common, as workers move to the city for work and return to village farms during the cultivation season. This can complicate efforts to ensure coverage of different rounds of treatment. Urban mobility may also impact the quality of population data and resulting epidemiological estimates. In Santo Domingo, Dominican Republic, for instance, population mobility resulted in out-of-date census data (impacting the coverage denominator) and in persons receiving treatment who resided outside the targeted area (impacting the numerator) [14]. 

Compared to rural areas, drug distributors may be less likely to find urban residents at home during the day given long work hours and commutes to workplaces that are sometimes located in other areas of the city [20]. The more repeat visits are required, the greater the cost implications. Challenges in fixing a suitable place and time for preventative treatment are compounded when the opportunity costs of participating vie with daily livelihood activities and further emphasize the need for flexible and context-specific delivery strategies. 

### 2.4. Trust in People and Institutions

The heterogeneity and mobility that characterizes urban areas also have implications for trust in people and institutions. The constant flow of migrants into and within urban areas in search of work and housing means that neighbors may be strangers. In these dynamic contexts, trust is difficult to build and can be quick to erode given the rapid manner in which misinformation can spread. These challenges are particularly relevant to NTD programming. In any setting, media content or rumors may result in misconceptions about side effects, leading to poor compliance and disinterest in MDA campaigns. However, given the population density and variety of media in cities, the speed at which rumors spread may be greater than rural settings [21]. The absence of trusted community leaders and the presence of program staff unfamiliar with the communities they are serving, may also amplify distrust in urban MDA. In Nepal’s Kathmandu Valley, adverse events in other parts of the country had a large effect on population compliance in the metropolitan area. In the years that followed, media continued to propagate the idea that MDA was unsafe, leading to widespread mistrust of the program and persistent low coverage [9]. MDA programs in Kenya, Haiti and India have also suffered when news outlets falsely attributed deaths due to MDA [22,23].

Without active efforts to anticipate or dispel misinformation, misconceptions and mistrust-- and sometimes even despite these efforts—mass public health campaigns in urban areas can be undermined. For example, in Kathmandu, researchers found that partial immunization in children was largely due to not preparing primary caretakers for possible side effects [24]. Perceived distrust in the quality and safety of drugs may also have a negative impact on both coverage and compliance [17,25] as demonstrated by Krentel et al.’s [17] study of MDA for the elimination of LF in Depok City, Indonesia. 

## 3. Urban MDA Strategies

Recognizing these urban specific challenges, adaptation of the core components of MDA (training, supervision, supply chain management and community engagement) may be needed to achieve coverage goals. Based on our narrative review five key adaptations were found helpful in urban settings:

### 3.1. Establishing Need

A first step is to establish whether an MDA campaign is necessary. Dividing the city into several smaller implementation units can help establish where in the city MDA is required and where it is not, a strategy referred to as micro-targeting. Identifying any populations or areas of the city that do not need treatment and can simplify the task, allow a more focused use of resources and significantly reduce programmatic costs. In Santo Domingo, Dominican Republic, for example, only the slum areas and neighboring apartments had to be targeted for treatment [14]. 

Some urban areas may not require treatment. Recent study suggests that MDA for LF may not be necessary in many West African cities [26] where levels of infection are low and where the disease is transmitted by *Culex* spp., much less effective vectors for *Wuchereria bancrofti* (the parasite causing LF) than the *Anopheles* spp. common in rural areas [26]. This finding, along with mapping surveys indicating that fewer than 1% of tested individuals in urban Abidjan and Conakry have detectable filarial antigen, suggested that vector control methods such as the use of long-lasting insecticidal nets and mosquito repellants, or environmental engineering techniques that reduce vector-breeding sites, may be feasible alternatives for LF control. Similarly, in high-risk areas of the city in close proximity to breeding sites (e.g., informal settlements), it may be cost effective to conduct xenomonitoring (measuring infection in the vector) studies to assess the presence of *W. bancrofti* within mosquitoes, before embarking on a 5- to 7-year MDA program [26].

Checking impact after several rounds of MDA is another potential strategy. For example, after six rounds of MDA with low coverage in the peri-urban areas of Kathmandu, Nepal, prevalence data were collected from three sentinel sites (previously selected at baseline) and another ten spot-check sites. This interim assessment indicated that MDA could be stopped in three out of the four implementation units [9]. 

### 3.2. Conducting Effective MDA Campaigns

Whether slums, peri-urban informal settlements, housing complexes, single-family homes, or high-rises—identifying the various populations groups and neighborhoods within each implementation unit (IU) is crucial in designing tailored strategies for community distribution. These groups will likely differ in how they can best be reached—what time of day or week, which platform, by whom and using which communication message(s). As a result, urban MDA may need to include multiple strategies to reach different populations. 

#### 3.2.1. Identifying Appropriate Distribution Platforms

The common distribution platforms for MDA are schools, door-to-door, and fixed posts/booths set up within the community at strategic locations. Fixed posts are particularly suitable in urban areas where door-to-door distribution is not feasible. For instance, in hard-to-access compounds or high-rise buildings, MDA booths at building or compound entrances are effective in reaching people when they are leaving or returning home. In other urban settings, a mix of distribution strategies may be appropriate. In Bamako, Mali, the NTD program used multiple approaches, including MDA in private and public schools, fixed post distribution at strategic locations (markets, major intersections, in front of chief of quarter residences and government offices), as well as door-to-door distribution in selected locations [27]. Likewise, in two urban districts in Indonesia, MDA was implemented simultaneously in government and private offices, at schools, in households as well as factories, in order to increase the participation of the working population [17]. In American Samoa, organizing MDA for LF in conjunction with church attendance was effective in facilitating the logistics of distribution and provided a culturally appropriate way to connect with urban communities [28]. Another option is to integrate MDA for NTDs into other delivery platforms (e.g., immunization programs), especially those that penetrate disadvantaged settings such as slums where healthcare infrastructure is limited. 

In order to more effectively reach working or otherwise mobile urban populations, NTD programs strategies have incorporated flexible MDA hours and multiple distribution sites or approaches. In Freetown, Sierra Leone, MDA was based on a rolling, dynamic model. The campaign was launched on a Thursday, allowing community health workers to reach schools and colleges. Distribution in prison populations, the military, council workers and the police was also carried out on Thursday and continued to Friday. The remaining population was targeted at mosques on Friday and churches on Sunday. On Monday, the commercial areas were covered and ‘mop-up’ activities began to reach households and areas that had been missed during the scheduled MDA [29]. The WHO Supervisor’s Coverage Tool has been used in some urban areas, such as in Haiti, to rapidly determine whether mop-up activities were necessary [29].

#### 3.2.2. Communication

Another important step in implementation of MDA for NTDs is communication, whereby key messages are widely conveyed–such as information on the specific NTDs and accompanying morbidity, how NTDs can be prevented, why an MDA is being undertaken, information on drug safety and possible side effects and the time and location of MDA. Communication campaigns emphasize the importance and benefits of treatment in advance of MDA with the purpose of fostering trust between the community and the campaign [25].

Particularly in urban areas, where NTDs may not be recognized as a threat, the health benefits of compliance are not always fully understood. NTDs may be ‘unseen’ if they are relatively uncommon, when infected people are asymptomatic and when outward signs of disease manifest themselves long after infection. Promoting the ‘non-health’ benefits of compliance is one strategy to overcome this challenge. In Indonesia, for instance, linking urban MDA campaigns with individual aspirations of being perceived as ‘modern,’ embracing non-traditional medicine, caring about family and community and being a good citizen who is acting on behalf of future generations and their country’s economic development, increased compliance [17,25]. According to Krentel et al., individuals were motivated to participate in MDA when family, friends and neighbors were also participating, resulting in the recommendation that future MDA messaging focus on the social norm of compliance and the safety of LF drugs globally and in Indonesia [17]. 

Even more so than in rural settings, the heterogeneity and diversity of the urban population—including a mix of highly educated, illiterate, minority and migrant groups who may speak different languages—means that strategies and information, education and communication (IEC) materials may need to be adapted to ensure participation by the entire population [9]. For example, to accommodate large numbers of internal migrants into the Kathmandu Valley, the Nepalese NTD program printed information materials in several local languages and used community centers to distribute information to specific ethnic groups and castes [9]. 

In urban areas, it may be helpful to vary the sites at which communication activities occur depending on the target group or day of the week. For example, messaging at schools, colleges, offices and factories can be conducted during weekdays, whereas street-based dissemination and announcements and leaflets distributed at mosques and churches can be undertaken on Fridays and weekends [25,29,30]. For urban communities to be effectively engaged, evidence suggests that it may be important to learn about MDA from multiple trusted sources, for example, health services, local leaders, household authority figures, the media and personal networks [25,29].

**Figure 1:**
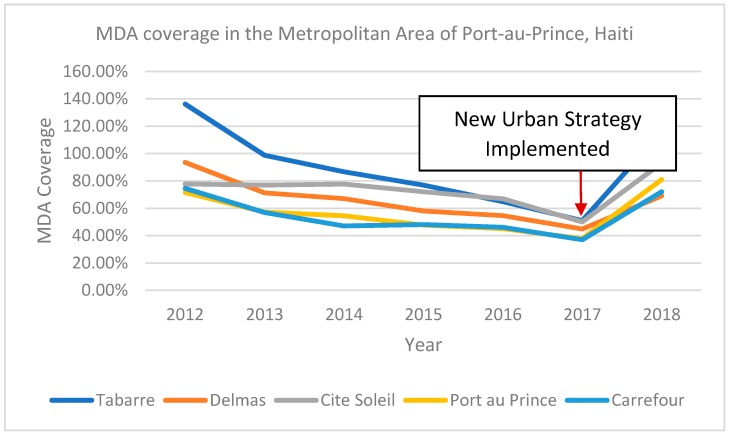
MDA coverage in the metropolitan area of Port-au-Prince, Haiti (2012–2018).

Use of multiple forms of popular media has proven particularly effective in urban areas [16,30,31]. These include local and national radio and TV spot messages, jingles and announcements at multiple times of the day, or during periods of high viewership or listenership, such as before evening news or sports programs [28]. Biritwum et al.’s study in Accra, Ghana found that 60–64% of urban study participants preferred being informed about MDA through the media and particularly the radio [16]. Traditional forms of broadcasting can be supplemented by testimonies, skits and programs where experts respond to questions and concerns from the public via phone-ins and short message service (SMS) [31]. News clips that show popular national figures and local celebrities such as artists and athletes participating in MDA have been employed effectively [31]. To reach the younger population, the use of the Internet and particularly social media may be beneficial [17,31]. Figure 1 describes a dramatic increase in MDA coverage in Port-au-Prince, Haiti following implementation of an intervention package that utilized multiple distribution and communication strategies tailored to the urban realities of that setting.

In the metropolitan area of Port-au-Prince, epidemiological coverage increased from 41% in 2017 to 80% in 2018 after implementing an intervention package tailored to this urban setting. Microplanning informed the positioning of distribution posts and the communication strategy was redesigned based on information from focus groups with adults and youth. The program held press conferences with high-level officials, showing them taking the pills, and testimony from lymphedema patients was broadcasted on TV and radio. The MDA team also used a FAQ sheet and created a hotline to provide information on adverse events and private physicians were involved throughout the campaign. The platform was adapted to increase the number of posts and MDA days and school-based distribution was strengthened. Real time coverage data was collected during the MDA using a mobile phone-based reporting system, and the Supervisor’s Coverage Tool was used to rapidly assess coverage and inform mop-up immediately after the MDA. Finally, the role of drug distributors was strengthened by increasing their visibility and official status with ID badges and T-shirts.

#### 3.2.3. Human Resources

**Figure 2:**
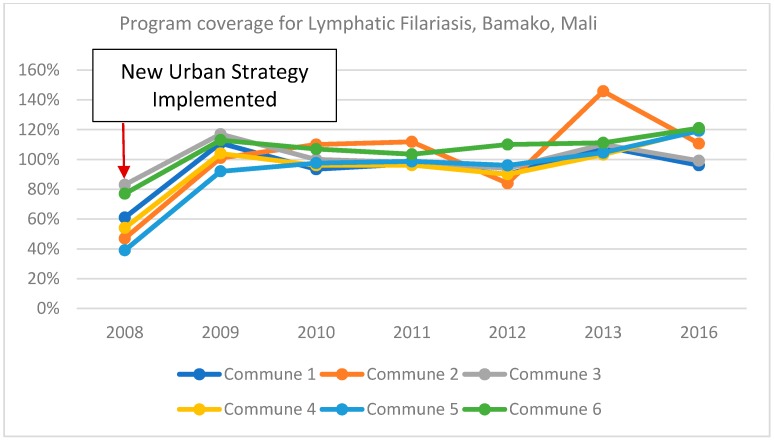
Program coverage for lymphatic filariasis in Bamako, Mali (2008–2016).

Attention to the heterogeneity of urban areas is also important in determining who should deliver treatment, the numbers needed and how workers are motivated and supported. Experience supports the value of matching MDA workers to different target populations such that trust and respect are maximized [19,25]. For example, based on their work in urban informal settlements in Kisumu, Kenya, Odhiambo, et al. recommended a community directed intervention (CDI) approach in urban, informal settlements, whereby community members decide who will distribute the drugs, how responsibilities will be allocated between MDA workers, as well as the most appropriate mobilization techniques, the timing of MDA and how drugs will be distributed [22]. Urban MDA campaigns in Bamako, Mali, identified nurses and nursing students as especially effective because their training left them better equipped to handle complex questions and allay any concerns about the MDA in a relatively well-educated population [9]. Figure 2 shows the increase in program coverage for LF that occurred as a result of this and other urban-specific adaptation in Bamako.

After implementing urban specific interventions in Bamako in 2008–2009, program coverage for LF increased from 60% to 106%. The revised strategy focused on strengthening the capacity of drug distributors by selecting people with higher levels of education (e.g., nurses) to distribute the drugs. In addition, communication messages directly addressed negative rumors that had been circulating thus improving community engagement. Finally, regional health staff worked closely with the Ministry of Education to strengthen monitoring of treatment in schools and improve scheduling and communication of planned activities. 

In an MDA campaign in Freetown, Sierra Leone, it was similarly observed that the more affluent areas of the city did not accept non-health workers as drug distributors, since they were regarded as less qualified [9]. The program changed its strategy the next year, hiring paid health workers to treat allocated areas within a 5-day period. Along with supportive supervision, this adaptation resulted in substantial improvements in treatment coverage.

Experience also indicates that MDA distributors who carry an official letter, display official identity badges, or wear campaign branded t-shirts, hats, or vests are better accepted in urban communities [14]. In the Dominican Republic, trust between the program and the community in Santo Domingo was strengthened by having local leaders select known and respected community members to serve as drug distributors and supervisors and providing them with an official identity card and ‘uniform’ [14]. Engaging local non-governmental organizations with strong ties in the community in the planning and administration of urban MDA has also been effective in building trust between the program and key leaders in the community, thereby improving the safety of drug distributors [9]. 

Finally, accurate projections of human resource requirements are essential given the population density and rapidly shifting demographics of urban settings. For example, in Niger’s capital Niamey, when door-to-door methods were employed in in the city’s densely populated ‘quartiers,’ drug distributors had to cover far more people, yet were initially paid the same daily rates as drug distributors in less populated settings. Adjustments were made based on these findings and both the number of and payments to distributors and supervisors in urban settings were increased [9]. 

#### 3.2.4. Managing Adverse Events

In urban contexts, where reports of adverse events (real or rumored) can spread rapidly due to the density of people and the variety of media sources, well managed communication strategies are critical. Ideally, plans to address adverse events—both medically and in the media—should be established prior to all urban MDAs. Similarly, responses to adverse events should be timely and decisive to reduce the development of negative impressions. Information, education and communication (IEC) campaigns may include media advocacy before and during the MDA to allay potential apprehensions. If possible, initial planning should involve private practitioners given their prevalence and importance as sources of health care in urban areas [9]. The national NTD program in Santo Domingo in the Dominican Republic, for example, trained health providers in advance of the MDA and requested that clinics be open during MDA to address any possible concerns [14]. 

## 4. Research Directions

Emerging from this review of urban-specific implementation challenges and adjustments to MDA processes is the need for further research that enables more effective and efficient NTD investments in urban areas. During the COR NTD panel on urban MDA in 2017 [31], researchers prioritized three major avenues of research: targeted mapping, context-specific methods to increase MDA coverage and monitoring and evaluation. 

### 4.1. Mapping–Identifying When and Where Interventions are Needed

While targeted mapping or remapping in urban areas may be a way to reduce unnecessary MDA, prior knowledge is needed about sub-populations or geographic units within urban areas, their respective risk for NTDs and how stable they are in time and space. Research of this nature can inform when, where and how often mapping activities might be indicated. Comparative cost assessments between more extensive mapping versus multiple rounds of MDA will also inform MDA decision making. The role of xenomonitoring in decisions on whether to start MDA for LF in urban areas is another issue requiring further research attention. 

In circumstances where mapping data is old, it may be important to assess the impact of other disease control efforts put in place since mapping was performed such as the uptake of insecticide treated bed nets and indoor residual spraying which target the vector that transmits LF.

Finally, mapping may also help determine the transmission potential of NTDs in urban and peri-urban areas and ensure that MDA activities are responsive to local realities. For example, if mapping identifies a high transmission potential for schistosomiasis in emerging peri-urban settings, differences in the availability of modern water management structures and school-based structures must be taken into account when planning NTD programs.

### 4.2. Methods to Increase Coverage of MDA

A second stream of research should investigate cost effective methods to ensure that MDA coverage targets in urban areas are met. This includes determining what approaches and platforms work best in urban settings (e.g., fixed post, door-to-door, markets, malls, schools, private pharmacies, other private health care settings, entrances to large high rises, workplaces, prisons, etc.), to achieve coverage targets and their relative cost.

The development of rapid assessment tools that identify the most successful, cost-effective, feasible, replicable service delivery package for specific urban contexts is another area of research that would allow for more tailored approaches to MDA planning and implementation. Similarly, methodologies that identify the different behavioral drivers of MDA participation in different urban micro-populations (educated vs. illiterate; rich vs. poor; etc.) and which solicit community inputs, would be helpful in crafting context-specific social mobilization messages. Of further interest is research on the relative effectiveness of these messages and the best modalities for communication, whether radio, billboards, television slots and so forth. Together, these data would inform the generation of micro-plans for urban areas and context specific strategies to improve MDA participation. Finally, the development of well-constructed case studies documenting the cost and impact of changing programmatic approaches will be valuable in demonstrating potential gains in adopting urban-sensitive approaches.

### 4.3. Monitoring and Evaluation

A final area of research relates to monitoring and evaluation of MDA in urban areas. Monitoring via surveys or routine data collection at the sub-district level is critical for diseases with elimination goals like LF where decisions to stop treatment are contingent on such information. However, these tools do not always provide accurate estimates in urban areas [32,33,34]. Challenges to measuring coverage in urban areas largely stem from population mobility and growth. Census estimates are quickly outdated and may lead to inaccurate denominators when calculating MDA coverage. Coverage surveys overcome some of these challenges but may leave out the same people missed during the MDA—again due to population mobility and daily, weekly and monthly migratory patterns. Novel methods to estimate coverage in urban settings that employ satellite data or cellular phone usage patterns to estimate population denominators, are potential solutions that need to be explored.

## 5. Conclusions

Considerable scale-up of NTD programs has occurred since 2007 but reaching people with preventive treatment has proven challenging in many rapidly urbanizing settings [18,20,35,36]. Achieving and sustaining MDA coverage in heterogeneous and dynamic urban populations requires careful planning of tailored approaches. 

Streams of research that could inform effective approaches to the control and elimination of NTDs in urban areas include understanding how and when to map or re-map urban areas, developing context-specific methods to motivate participation of urban residents in MDA and innovations in monitoring and evaluation. This research agenda is critical given the exponential growth of urban populations in low and middle-income countries and the rising social and health inequalities that rapid, unplanned urbanization brings [37,38]. The implications of urban growth for the control of NTDs and the manner in which they interact and overlap with other urban health issues, including non-communicable diseases, must also be considered [2]. Our ability to identify and address health challenges specific to urban areas will increasingly determine the overall health of populations as well as the success of current and future disease control programs–for NTDs as well as other emerging diseases.
